# Variable Expression of Neural Cell Adhesion Molecule Isoforms in Renal Tissue: Possible Role in Incipient Renal Fibrosis

**DOI:** 10.1371/journal.pone.0137028

**Published:** 2015-09-01

**Authors:** Jasmina Marković-Lipkovski, Maja Životić, Claudia A. Müller, Björn Tampe, Sanja Ćirović, Jelena Vještica, Nada Tomanović, Michael Zeisberg, Gerhard A. Müller

**Affiliations:** 1 Institute of Pathology, Medical Faculty, University of Belgrade, Belgrade, Serbia; 2 Section for Transplantation Immunology and Immunohematology, ZMF, University Medical Clinic, Eberhard Karls University, Tübingen, Germany; 3 Department of Nephrology and Rheumatology, Göttingen University Medical Center, Georg-August University, Göttingen, Germany; National Cancer Institute, UNITED STATES

## Abstract

Rare neural cell adhesion molecule (NCAM) positive cells have been previously described within the normal human adult kidney interstitium, speculating that they could increase in the interstitium with incipient interstitial renal fibrosis (IRF). In the present study, among 93 biopsy samples of various kidney diseases, NCAM^+^ interstitial cells were detected in 62.4% cases. An increased number of NCAM^+^ cells was significantly observed only in incipient IRF compared to normal renal tissues and advanced IRF stages (p<0.001), independently of underlying diseases (p = 0.657). All three major NCAM isoforms’ RT-PCR bands were visible either in normal or in kidneys with incipient IRF, albeit their mRNA expression levels measured by qRT-PCR were different. Applying qRT-PCR on pure NCAM^+^ cells population, obtained by laser capture microdissection, significant mRNA over-expression of NCAM^140kD^ isoform was found in NCAM^+^ cells within incipient IRF (p = 0.004), while NCAM^120kD^ and NCAM^180kD^ isoforms were not changed significantly (p = 0.750; p = 0.704; respectively). Simultaneously, qRT-PCR also showed significant αSMA (p = 0.014) and SLUG (p = 0.004) mRNAs up-regulation within the NCAM^+^ cells of incipient IRF, as well as highly decreased matrix metalloproteinases (MMP) -2 and -9 mRNAs (p = 0.028; p = 0.036; respectively). However, using double immunofluorescence MMP-9 could still be detectable on the protein level in rare NCAM^+^ cells within the incipient IRF. Further characterization of NCAM^+^ cells by double immunofluorescent labeling revealed their association with molecules involved in fibrosis. Fibroblast growth factor receptor 1 (FGFR1) and α5β1 integrin were extensively expressed on NCAM^+^ cells within the incipient IRF areas, whereas human epididymis protein-4 (HE4) was found to be present in few NCAM^+^ cells of both normal and interstitium with incipient fibrosis. Heterogeneity of NCAM^+^ interstitial cells in normal and incipient IRF, concerning molecules related to fibrosis and variable expression of NCAM isoforms, could suggest diverse role of NCAM^+^ cells in homeostasis and in regulation of renal fibrosis in diseased kidneys.

## Introduction

In addition to rare fibroblasts, scarce neural cell adhesion molecule (NCAM) positive cells with spindle shaped or dendritic morphology can be detected within the interstitium of the normal adult human kidney [1, 2]. These cells seemed to have arisen from metanephric mesenchymal cells expressing NCAM during kidney development, and selectively persist within the renal interstitium after birth [3]. Previously, it has been suspected that in early phases of repairing processes of a damaged kidney interstitial NCAM+ cells could increase [2, 4]. The origin of such NCAM^+^ cells in fibrogenesis or kidney repair and their relation to fetal NCAM^+^ mesenchymal cells is still unknown and remains to be clarified, as well as their pathophysiological significance.

It is well known that human renal interstitium, especially under fibrotic conditions, exhibits highly heterogeneity, mostly with regard to molecular markers expressed by interstitial cells [5]. Since NCAM is one of the receptors essential during kidney organogenesis [3], we would like to clarify whether the increase of NCAM^+^ interstitial cell lineage during kidney repair could differ from rare NCAM^+^ cells situated within normal renal interstitium and if they could share some of the markers involved in tissue wound healing processes, either those which contribute or those which could ameliorate fibrosis.

Fibroblast growth factor receptor 1 (FGFR1) is involved in fibroblast activation and proliferation that can be activated by different ligands including NCAM [6, 7]. Activation of FGFR1 by NCAM interaction additionally promotes FGFR1 recycling, resulting in sustained FGFR1 signaling that is important for fibroblast migration [8, 9]. Besides FGFR1, α5β1 integrin also cooperates in cell adhesion, proliferation and differentiation and plays a role in extracellular matrix assembly [10–13]. It has been shown that α5β1 integrin expressed by fibroblasts promotes acquisition of a myofibroblastic phenotype (activated fibroblasts with a typical α-SMA expression pattern), which constitute the dominant interstitial cells in a pro-fibrotic microenvironment [1, 13, 14]. Another up-regulated gene in myofibroblasts is human epididymis protein-4 (HE4), also found in non-α-SMA expressing cells [15]. HE4 is a protein which suppresses the activity of multiple proteases, including serine proteinase and matrix metalloproteinases, and inhibits their capacity to degrade type I collagen [16]. Matrix metalloproteinases -2 and -9 (MMP-2 and MMP-9) are mainly linked to degradation of collagen I which expression is regulated by SLUG and SNAIL transcription factors [17, 18].

Thus, we felt encouraged to investigate whether NCAM^+^ interstitial cells could share aforementioned molecules involved in fibrosis and/or extracellular matrix remodeling and some of the transcriptional factors which regulates their expression. Additionally, molecules which could counteract the damaging signal such as BMP7 and its ALK3 receptor [19, 20], as well as molecules which rapidly increase under hypoxic conditions such as erythropoietin (EPO) [5, 21], were also explored in NCAM^+^ interstitial cells for the characterization of their heterogeneity and function. Presence of NCAM^+^ interstitial cells was examined in various kidney diseases, correlating their increase to the severity of interstitial fibrosis. Moreover, known major NCAM isoforms (120kD, 140kD, and 180kD), were investigated in kidneys with fibrosis and compared to their presence and mRNA expression levels in normal kidneys.

## Materials and Methods

### Tissue specimens

All study participants or their close relatives (in the cases of cadaveric kidney tissues) gave the written informed consent to participate in the study, which was approved by Institutional Review Board of the Faculty of Medicine, University of Belgrade (reference number 29/VI-7). Informed consent included agreement to use the rest of the tissue (after diagnostic work-up) for scientific purposes.

Normal renal tissues were obtained from 10 cadaveric kidneys that were not transplanted, as well as from 10 non-tumor renal tissues obtained after nephrectomies due to renal tumors. 93 kidney biopsy specimens were included in this study after routine diagnostic: lupus nephritis (LN)—25 cases, focal-segmental glomerulosclerosis (FSGS)—18 cases, membranous glomerulonephritis (MGN)—13 cases, membrano-proliferative glomerulonephritis (MPGN)—8 cases, renal graft—9 cases, IgA nephropathy—6 cases, mesangio-proliferative glomerulonephritis (MesPGN)—5 cases, rapid-progressive glomerulonephritis (RPGN)– 5 cases, minimal change disease—4 cases.

Renal tissue from the first core needle biopsy of each patient was formalin-fixed, paraffin-embedded, routinely stained with H&E, PAS, silver methenamine and Masson trichrome, and further used for immunohistochemistry. The second renal biopsy core was put into cell culture medium RPMI 1640 (PAA Laboratories GmbH, Austria) immediately after removal, snap-frozen in liquid nitrogen, used for routine immunofluorescent diagnosis, and the rest of the tissue stored at -80°C for further immunostaining. In the cases where we had enough tissue, a piece of tissue sample was conserved in RNAlater (Qiagen Ltd., Hilden, Germany), a RNA stabilization reagent, for subsequent efficient reverse transcriptase PCR (RT-PCR) analysis.

### Immunohistochemistry

Immunostaining was applied on paraffin and cryostat sections. Paraffin sections were treated by microwave for 20 min at 400W in citrate buffer (pH 6.0) after deparaffinization and hydratation. Five μm thick frozen sections cut from each tissue were fixed in acetone for 10 min, air-dried at room temperature for 1 hour. After antigen retrieval both frozen and paraffin samples were incubated for 1 hour at room temperature with primary antibody: NCAM clone Eric-1 (1:100, Ancell Corporation, USA), NCAM clone 123C3.D5 (LabVision, USA), FGFR1 clone M19B2 (1:100, Abcam, UK), HE4 (1:40, ab24480, Abcam, UK), integrin α5β1 clone SAM-1 (1:200, Chemicon Europe). NCAM/Eric-1 was used on cryostat samples, while paraffin samples were incubated with NCAM/123C3.D5. The EnVision staining method (DAKO, Denmark), visualization of antigen-antibody reaction by 3,3'-diaminobenzidine (DAB) and subsequent counterstaining with hemalaun (Merck, USA), were conducted. Controls were performed as previously described [2], and for mouse monoclonal antibodies as isotype control mouse IgG1 (ab91353, Abcam, UK) antibody was also used. Slides were evaluated using the light microscope BX53 with DP12 CCD camera (Olympus, Germany).

### Assessment of interstitial renal fibrosis and number of NCAM positive cells

Examining the relationship of NCAM^+^ interstitial cells with degree of interstitial renal fibrosis (IRF), immunostaining was performed on paraffin sections and evaluated by light microscopy, as a number of positive cells per field of view on the magnification x400 in the region with the most extensive interstitial NCAM positivity in a biopsy core. Degree of IRF was semi-quantitatively assessed on biopsies stained with PAS and Massone-trichrome, applying a scale from 0 to 3 with 0 meaning no IRF, 1—less than 25% of renal tissue with IRF (marked as incipient IRF), 2–25% to 50% of renal tissue with IRF, and 3—more than 50% of renal tissue with IRF cells. 20 cases of normal renal tissue were also assessed for the number of NCAM positive cells within the interstitium. All assessments were done independently by two pathologists.

### Double immunofluorescence labeling

Five μm-thick cryostat sections were treated as previously described [2]. In order to obtain double fluorescent labeling NCAM/granzyme B, NCAM/FGFR1, NCAM/integrin α5β1, NCAM/αSMA cells and NCAM/MMP9, we applied rabbit monoclonal antibody against NCAM clone EP257Y (1:200, Epitomics, UK) followed by Cy3- conjugated goat anti-rabbit antibody (1:2000, Dianova), and mouse monoclonal antibodies against granzyme B (2C5) (sc-8022, Santa Cruz Biotechnology Inc., 1:50) or FGFR1 (clone M19B2, 1:100) or integrin α5β1 (clone SAM-1, 1:200) or αSMA clone 1A4 (1:400, Dako, Denmark) or MMP9 (1:100, Calbiochem, USA) were added followed by goat anti-mouse IgG-Alexa 488 (1:1000, Invitrogen). For NCAM/HE4 and NCAM/EPO cells detection, mouse monoclonal NCAM/Eric-1 antibody (1:100) followed by goat anti-mouse IgG-Alexa 488, and rabbit polyclonal HE4 antibody (1:100, ab85179, Abcam, UK), as well as rabbit polyclonal anti-EPO (1:100, ab126876, Abcam, UK), followed by Cy3-conjugated goat anti-rabbit antibody were applied. Nuclei were identified by 4,6-diamino-2-phenylindolyl-dihydrochloride (DAPI; 1 μg/ml). Controls were performed in all experiments as previously described [2]. Sections were mounted with Fluoro Preserve Reagent (Calboichem, Germany). Slides were analyzed either on LSM 510 Confocal Microscope with Apotome (Carl Zeiss, Germany) using the AxioVision Release 4.8.2 (Carl Zeiss, Germany) software version for analysis and documentation, or on epifluorescence microscopy with F-View CCD camera (Olympus, Germany), whereby digital pictures of each fluorescence channel were taken and superimposed for the specific antibody staining using the software AnalySIS from Soft Imaging Systems (Olympus) as previously published [2, 22].

### Isolation of RNA and reverse transcription PCR

Isolation of RNA from renal tissues and reverse transcription PCR (RT-PCR) procedures for detection of specific NCAM isoforms were performed as previously described in details [22]. For RT-PCR analyses, β-actin was used as a housekeeping gene.

### Laser capture microdissection, RNA isolation and quantitative real-time reverse transcription PCR

Frozen renal tissues obtained from normal kidneys and from biopsies with incipient IRF were stained with NCAM/Eric-1 antibody. Only NCAM^+^ cells localized within renal interstitum exhibiting dendritic morphology were marked for catapulting in laser capture microdissection (LCM) procedure. Between 35 and 50 NCAM^+^ cells were captured from each sample using PALM MicroBeam (Zeiss, Germany) and afterward stored on -80°C in microtubes with 100μl RNAlater reagent until further analysis.

RNA isolation was carried out using Arcturus PicoPure RNA isolation kit (Applied Biosystems, Germany), suitable for high quality RNA extraction from small samples. The quality of the isolated RNA was assessed using NanoDrop 2000 spectrophotometer (Thermo Scientific, Germany). 50 ng of total RNA was digested with DNaseI (Sigma) and used for cDNA synthesis using SuperScript II Reverse Transcriptase (Life Technologies). For quantitative real-time reverse transcription PCR (qRT-PCR) analysis, diluted cDNA (1/10) was used as a template in a Fast SYBR Green Master Mix (Life Technologies, Germany) and run in StepOnePlus Real-Time PCR System (Applied Biosystems) in a total reaction volume of 20 μL. Primers were designed and purchased from PrimerDesign. Primer sequences are shown in Table 1. Samples were run in triplicates and the mRNA expression levels were quantitatively analyzed and normalized to the level of glyceraldehyde 3-phosphate dehydrogenase (GAPDH) housekeeping gene. Before decision to use GAPDH as housekeeping gene in qRT-PCR procedure, we also tested 18s and β-actin. As signals for GAPDH were the most consistent within the analyzed samples we used it further analyses in qRT-PCR. GAPDH primers are also provided by PrimerDesign, but the sequences are undisclosed.

**Table 1:** Primer sequences used for qRT-PCR procedures.

	Forward primer 5’ to3’	Reverse primer 5’ to3’	ProductSize
**NCAM-120**	GAACCTGATCAAGCAGGATGACGG	CTAACAGAGCAAAAGAAGAGTC	321 bp
**NCAM-140**	GTCCTGCTCCTGGTGGTTGTG	CCTTCTCGGGCTCCGTCAGT	264 bp
**NCAM-180**	CGAGGCTGCCTCCGTCAGCACC	CCGGATCCATCATGCTTTGCTCTC	336 bp
**FGFR1**	GGCTACAAGGTCCGTTATGCC	GATGCTGCCGTACTCATTCTC	105 bp
**WFDC2 (HE4)**	AGAACTGCACGCAAGAGTG	TTGAGGTTGTCGGCGCATT	52 bp
**α-SMA**	AAGCACAGAGCAAAAGAGGAAT	ATGTCGTCCCAGTTGGTGAT	76 bp
**SLUG**	ACTCCGAAGCCAAATGACAA	CTCTCTCTGTGGGTGTGTGT	119 bp
**SNAIL**	GGCAATTTAACAATGTCTGAAAAGG	GAATAGTTCTGGGAGACACATCG	105 bp
**MMP2**	TACAGGATCATTGGCTACACACC	GGTCACATCGCTCCAGACT	90 bp
**MMP9**	TGTACCGCTATGGTTACACTCG	GGCAGGGACAGTTGCTTCT	97 bp
**ALK3**	GGACATTGCTTTGCCATCATAG	GGGCTTTTGGAGAATCTTTGC	112 bp
**BMP7**	CCTCCATTGCTCGCCTTG	TATGCTGCTCATGTTTCCTAATAC	114 bp

### Statistical analysis

Statistical analysis was performed using the IBM SPSS software, version 20.0. Each numerical variable was tested for normality of distribution, using Shapiro–Wilk and Kolmogorov–Smirnov tests, as well as considering skewness and kurtosis before the decision of implementation parametric or nonparametric statistical tests. For the assessments of numerical data between two groups, Student t-test or Mann-Whitney U were applied depending on the normality of data distribution. ANOVA was used for the assessment of numerical data among more than two groups, only if Levene’s test of homogeneity of variances allowed it (p>0.050), otherwise Kruskall-Wallis test, followed by Mann-Whitney U were applied. *P* values <0.05 were considered to be significant.

## Results

### NCAM positive cells were significantly increased in incipient renal interstitial fibrosis

**Fig 1:**
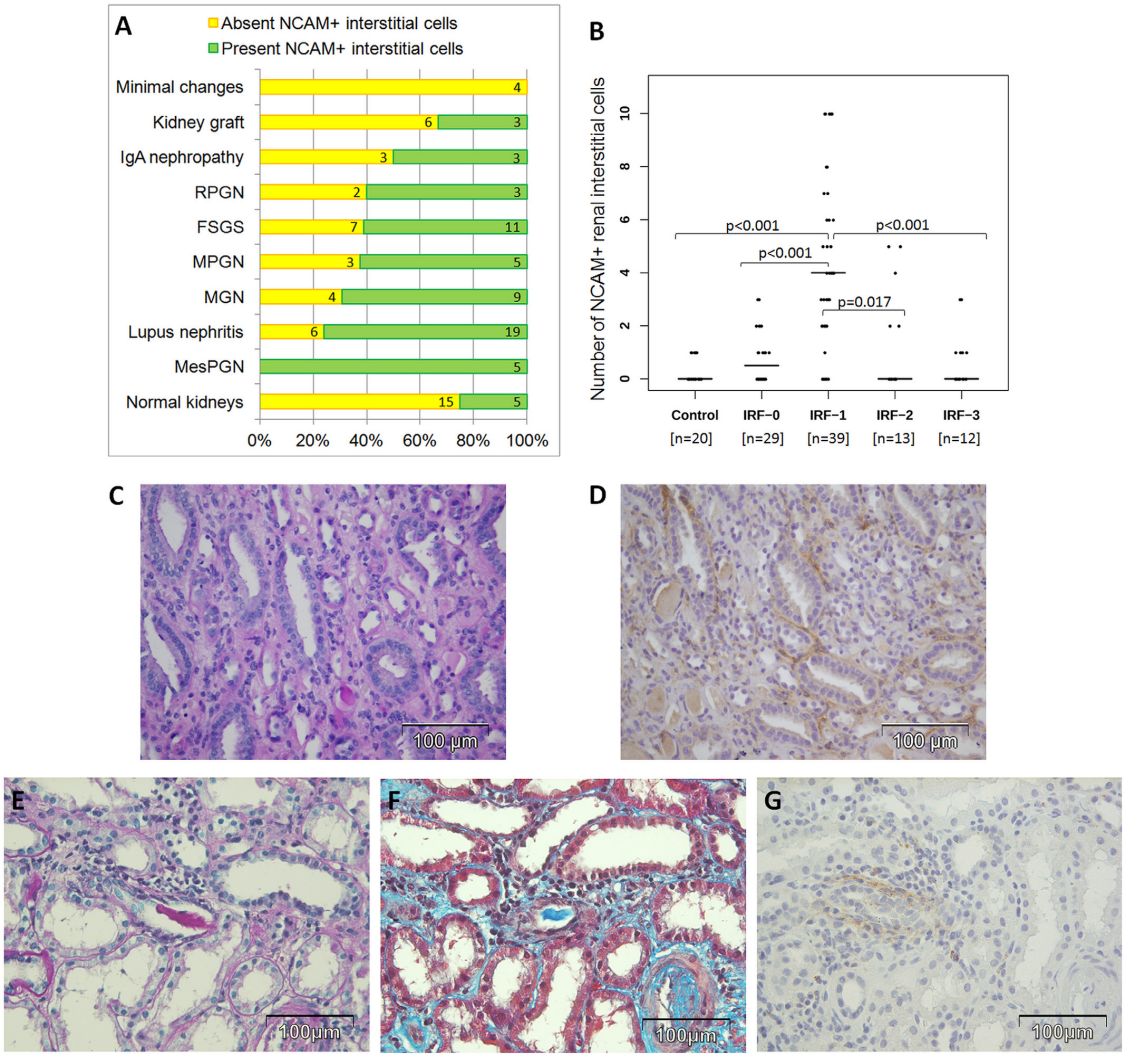
NCAM positive interstitial staining among various kidney diseases and their relationship to severity of renal interstitial fibrosis. **(A)** Frequency of interstitial NCAM positivity among various kidney diseases. **(B)** Number of detected NCAM^+^ cells per field of view on ×400 magnification in controls and in diseased kidneys with regard to severity of interstitial renal fibrosis (IRF); p values after applying Mann-Whiteny U test. **(C-D)** FSGS with slight interstitial fibrosis (IRF-1) without tubular atrophy exhibiting an increased diffuse NCAM interstitial positivity detected on slides from paraffin-embedded tissue. (C) PAS, x400. (D) Immunoperoxidase staining, NCAM clone 123C3.D5, x400. **(E-G)** Lupus nephritis with NCAM positive interstitial cells detected focally around tubuli in the area with slight IRF (IRF-1). (E) PAS, x400. (F) Massone trichrome staining, x400. (G) Immunoperoxidase staining, NCAM clone 123C3.D5, x400.

Renal interstitial NCAM^+^ cells were rarely present in the tubulointerstitial compartments of normal human kidneys, used as a control group (Fig 1A). However, in 93 paraffin-embedded biopsy specimens with various degrees of interstitial fibrosis, NCAM^+^ interstitial cells were seen in 58 cases (62.4%). NCAM^+^ interstitial cells were detected in 100% of MesPGN, 76.0% of LN, 69.2% of MGN, 62.5% of MPGN, 61.1% of FSGS, 50% of IgA nephropathy, 33.3% of renal grafts, 25% of RPGN, while NCAM^+^ interstitial cells were not detected in 4 cases of minimal change disease (Fig 1A). Mean number of NCAM^+^ cells were significantly higher in diseased kidneys (mean 2.45 NCAM^+^ cells, 95% CI (1.83–3.07)) compared to controls (mean 0.25 NCAM^+^ cells, 95% CI (0.4–0.46)), t = 6.731; p<0.001. A statistically significant increase of NCAM^+^ interstitial cells was present in incipient IRF, assessed as scale 1, compared to all others scales of fibrosis independently of the pathohistological diagnosis, as it is presented on Fig 1B. Relationship between number of NCAM^+^ cells and underlying kidney diseases classified according to IRF stages was further analyzed (Table 2), however, there were no significant differences. These data support our previous findings that increase of interstitial NCAM^+^ was independent of diagnosis, but depends only on extent of interstitial fibrosis, appearing almost exclusively in early stages (IRF-1), as illustrated in Fig 1B.

**Table 2:** Distribution of diagnosis and neural cell adhesion molecule (NCAM) interstitial positivity (including the number of NCAM positive interstitial cells presented with mean ±SD) observed among stages of interstitial renal fibrosis (IRF).

Diagnosis	IRF-0		IRF-1		IRF-2		IRF-3	
	Total N (NCAM+ cases)	mean*±SD	Total N (NCAM+ cases)	mean*±SD	Total N (NCAM+ cases)	mean*±SD	Total N (NCAM+ cases)	mean*±SD
**FSGS**	6 (3/6)	0.8±1.2	11 (8/11)	3.6±3.6	1 (0/1)	0.0±NA	-	-
**Kidney graft**	-	-	3 (2/3)	3.0±3.0	1 (1/1)	2.0±NA	5 (0/5)	0.0±0.0
**MGN**	7 (4/7)	1.0±1.2	4 (4/4)	6.5±4.4	-	-	2 (1/2)	1.5±2.1
**Lupus nephritis**	5 (2/5)	0.6±0.9	13 (12/13)	5.3±3.3	6 (4/6)	3.5±3.8	1 (1/1)	1.0±NA
**MesPGN**	1 (1/1)	2.0±NA	4 (4/4)	3.8±1.7	-	-	-	-
**MPGN**	2 (1/2)	0.5±0.71	3 (3/3)	3.7±2.1	3 (1/3)	1.7±2.9	-	-
**Minimal changes**	4 (0/4)	0.0±0.0	-	-	-	-	-	-
**IgA nephropathy**	4 (2/4)	0.5±0.6	1 (1/1)	4.0±NA	1 (0/1)	0.0±NA	-	-
**RPGN**	-	-	-	-	1 (0/1)	0.0±NA	4 (3/4)	0.8±0.5
Statistical analysis	-	p = 0.527^#^	-	p = 0.657^#^	-	p = 0.831^#^	-	p = 0.137^##^

N- number of cases;

*- mean number of NCAM+ cells per field of view x400;

NA- not applicable;

^#^- ANOVA test was applied;

^##^- Kruskall-Wallis test was applied because Levene’s test of homogeneity of variances was <0.050 and consequently ANOVA could not be used.

Fig 1C illustrates routine PAS staining with diffuse incipient renal fibrosis (IRF-1) of patient with FSGS. Applying immunohistochemical staining, within the same area many peritubular NCAM^+^ cells were detected in the interstitial compartment (Fig 1D). However, NCAM^+^ interstitial cells were usually detected focally around tubuli in the area with slight IRF (IRF-1), as it is shown in Fig 1E–1G.

**Fig 2:**
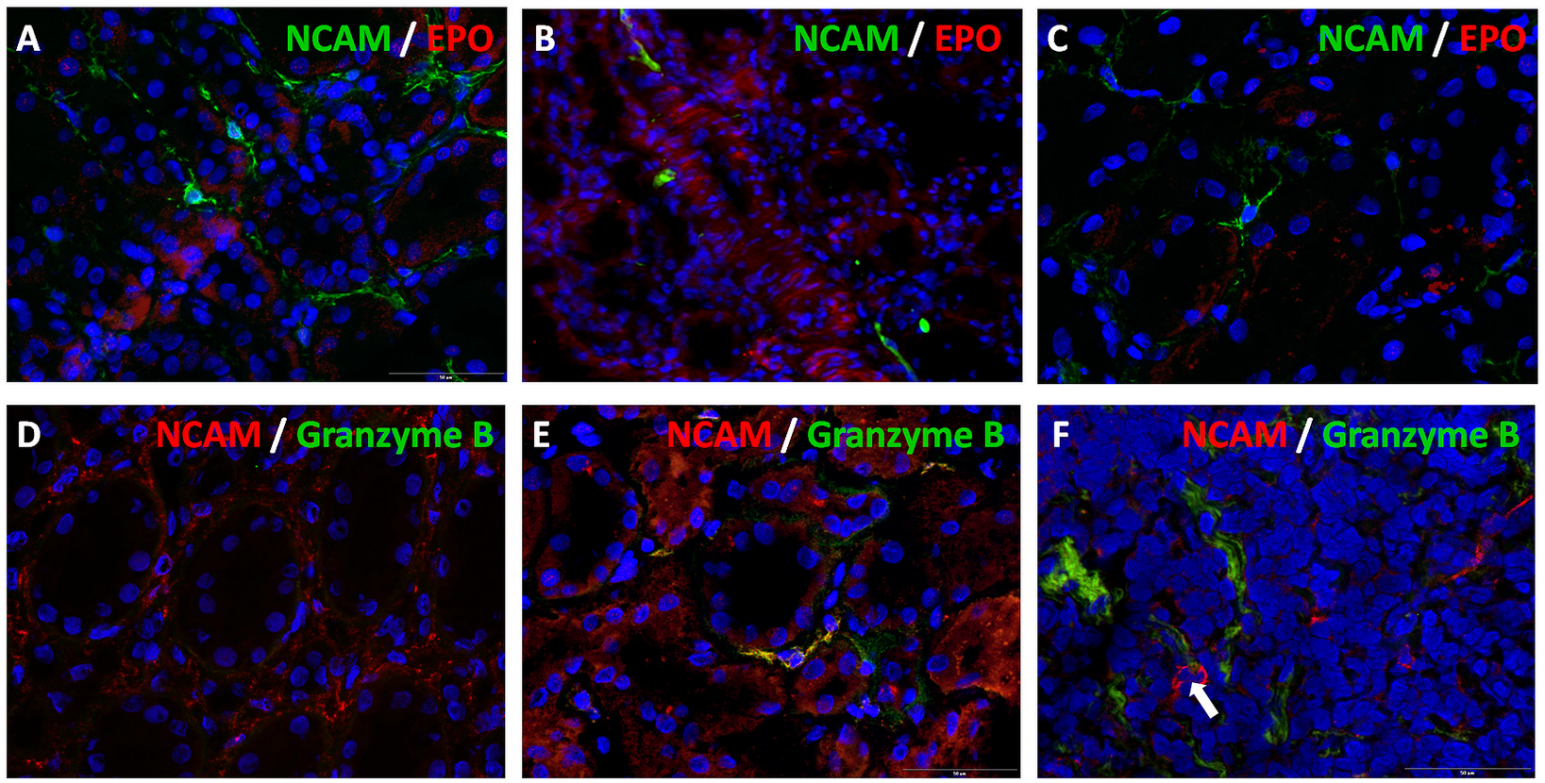
Double immunofluorescent labeling of NCAM with erythropoietin (EPO) and granzyme B. **(A-C)** NCAM positive interstitial cells did not express EPO. Merge of NCAM (clone Eric-1) and EPO, cryostat sections, double immunofluorescent labeling, x400. **(D)** Diffuse NCAM (clone EP257Y) staining in peritubular incipient interstitial fibrosis of FSGS case, without any granzyme B positivity, cryostat section, double immunofluorescene, x400. **(E)** Overlapping of NCAM (clone EP257Y) and granzyme B in a single cell within the whole biopsy core of the case illustrated in previous picture, cryostat section, double immunofluorescence, x400. **(F)** Mononuclear interstitial inflammatory infiltrate of lupus nephritis, arrow indicates two NCAM+ cells without overlapping with granzyme B, cryostat section, double immunofluorescence, x400.

In order to see whether NCAM positive cells could represent a population of erythropoietin (EPO) producing cells we further performed double immunolabeling but unfortunately we could not succeed to obtain immunofluorescent staining for EPO producing cells (Fig 2A–2C). Since NCAM can be also expressed by natural killer (NK) cells of the innate immune system, double NCAM/granzyme B immunostaining was performed to clarify their relationship in incipient renal fibrosis. In the Fig 2D, an area of diffuse NCAM interstitial staining is visible without any granzyme B positivity. Within the whole biopsy sample of the same case that belongs to FSGS with incipient IRF only a single interstitial cell expressed both NCAM and granzyme B (Fig 2E). Among cases of lupus nephritises, overlapping between these two molecules has not been detected, even within areas of mononuclear interstitial infiltrates (Fig 2F). Thus, there were interstitial NCAM^+^ cells different from NK cells that were almost exclusively increased in incipient renal fibrosis.

### All NCAM isoforms were detected in normal kidney and incipient renal fibrosis

**Fig 3:**
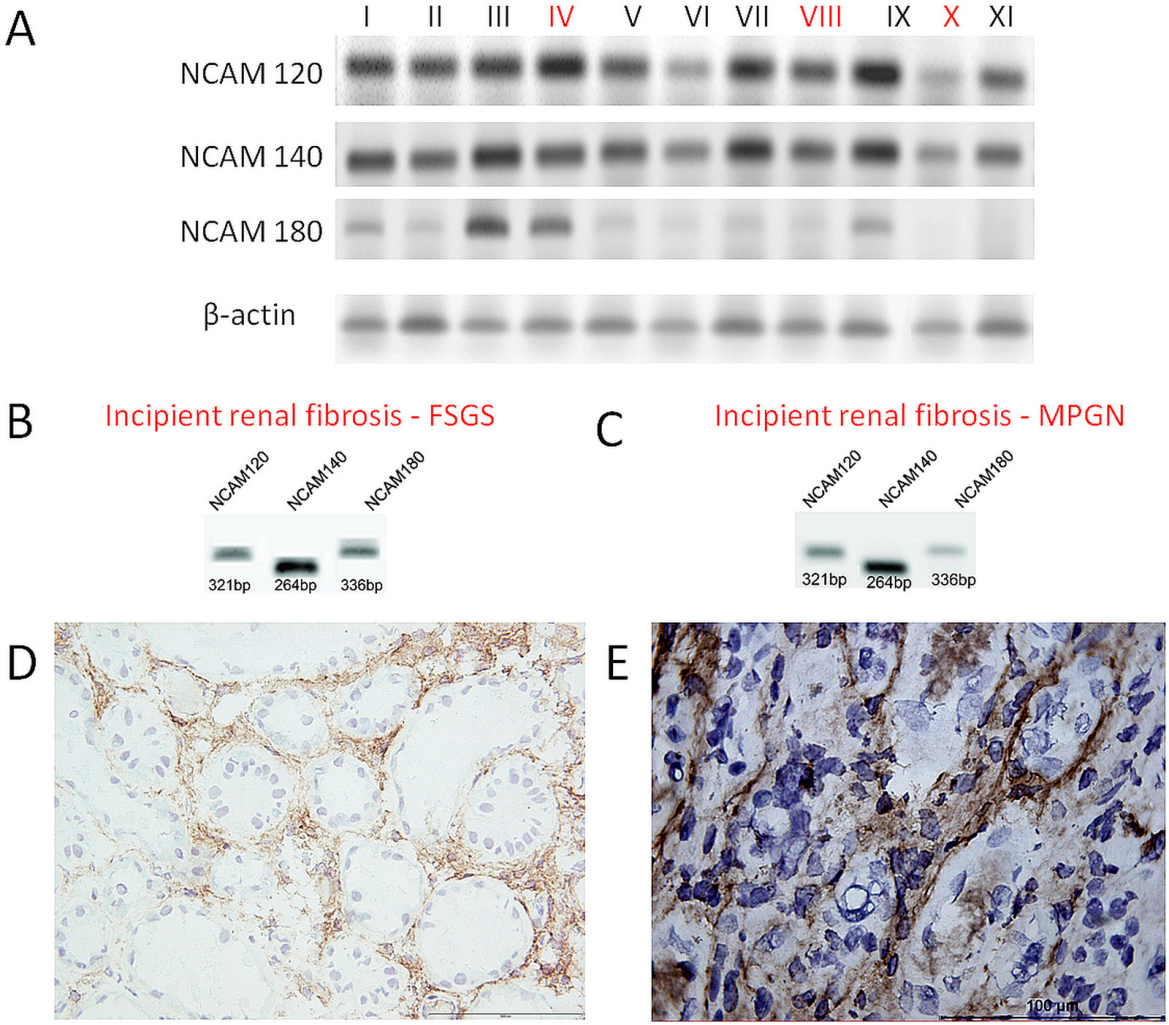
Presence of NCAM and its isoforms in normal and fibrotic kidneys. (**A**) RT-PCR: three NCAM isoforms in different renal samples, fibrosis was present in 3 cases, lanes IV, VIII and X. (**B**) RT-PCR: presence of all NCAM isoforms in FSGS. (**C**) RT-PCR: presence of all NCAM isoforms in MPGN. (**D**) Same case as Fig (B): increased NCAM expression in areas with slight fibrosis on cryostat section, immunoperoxidase, clone Eric-1, x200. (**E**) Same tissue as Fig (C): NCAM positivity in peritubular incipient interstitial fibrosis shown on cryostat section, immunoperoxidase, clone Eric-1, x400.

Considering that the three major NCAM isoforms could not be differentiated by immunohistochemical staining, we further proceeded RT-PCR analyzes on normal and fibrotic tissue lysates in order to detect presence of specific isoforms. The mRNA expression of all NCAM isoforms was detected in eight control renal tissues and five cases with incipient IRF (Fig 3A: lanes IV, VIII and X; Fig 3B and 3C). All three major isoforms were found to be expressed both in normal and kidneys with incipient IRF. The same cases were also stained for NCAM, using cryostat sections, and two cases are presented on Fig 3: FSGS with focal incipient fibrosis (Fig 3D) and results of RT-PCR (Fig 3B), and MPGN with incipient fibrosis (Fig 3E) and results of RT-PCR (Fig 3C). We need to underline that NCAM staining was more intensively visible on these cryostat sections (Fig 3D and 3E) than on slides from paraffin-embedded tissues (Fig 1) that were used in our study for the assessments of NCAM positivity in various kidney diseases with variable degree of IRF (Fig 1).

### NCAM^140kD^ isoform was overexpressed in NCAM positive renal interstitial cells within areas of incipient renal fibrosis

**Fig 4:**
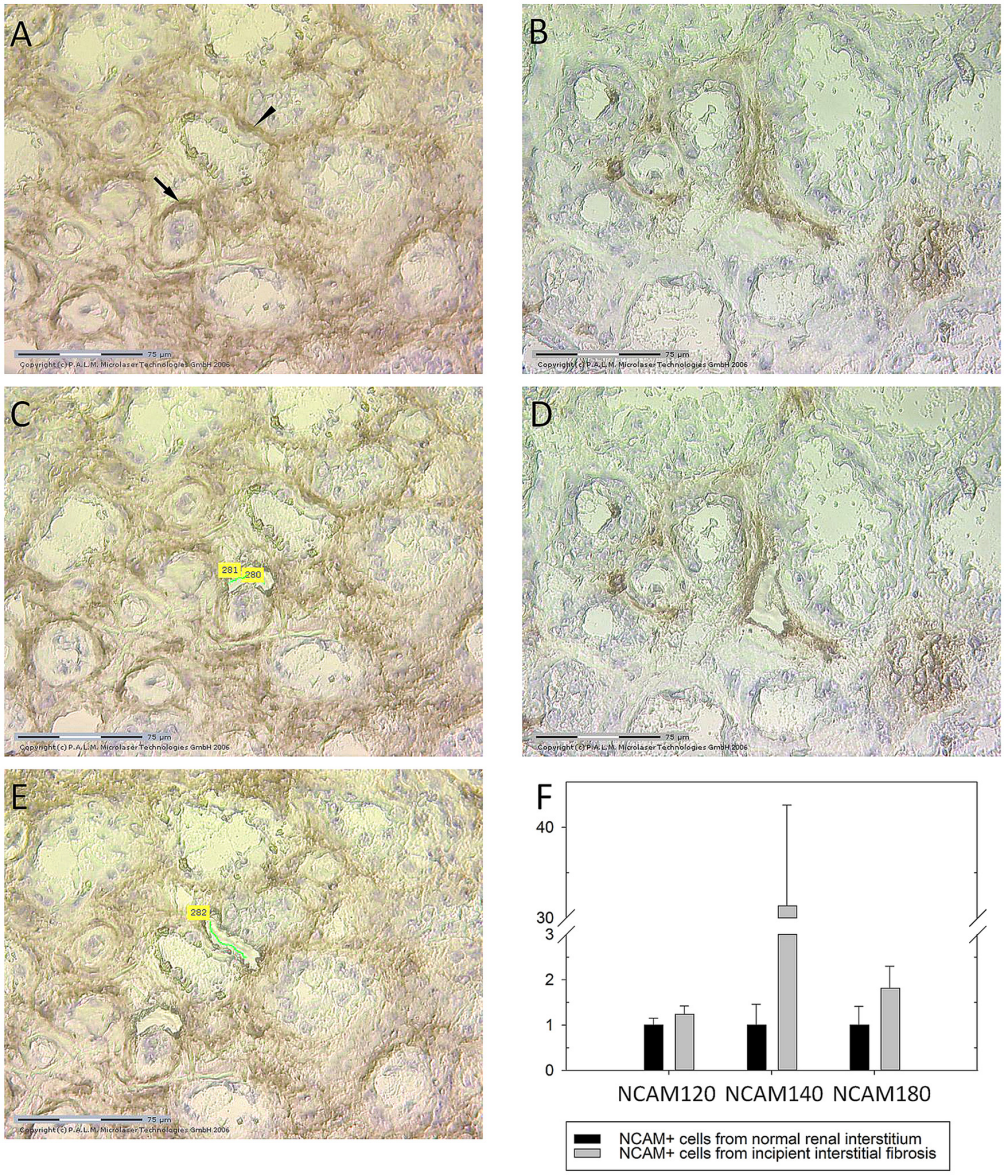
Isolation of NCAM positive renal interstitial cells by laser capture microdissection (LCM) and changes in relative mRNA NCAM isofroms expression levels in incipient renal fibrosis. **(A)** Slide performed on cryostat section and stained by NCAM, clone Eric-1, with widespread NCAM expression, prior laser capture microdissection (arrow indicates the first selected NCAM positive cell for further LCM, while arrowhead shows second selected area). **(B)** Slide with rare NCAM cells within normal interstitium prior LCM. **(C), (D)** and **(E)** the same slides as Fig (A) and (B) after LCM procedure. **(F)** Relative expression levels of NCAM mRNAs isoforms, determined by quantitative real-time PCR (qRT-PCR), in NCAM^+^ cells captured by LCM from normal and from renal tissue with incipient IRF, data are presented with mean values and standard error bars; due to high variability of variables, exclusively in diseased kidneys, nonparametric Mann Whitney U test was applied to assess the difference in mRNA levels between controls and diseased kidneys; there were 6 samples (2 cases in triplicates) of control cases and 42 (14 cases in triplicates) samples of cases with incipient renal fibrosis.

Since tissue lysates could contain other NCAM expressing cells, not only the interstitial spindle shaped NCAM^+^ cells which were within the focus of our study, we decided to perform laser capture microdissection (LCM) which allowed us to separate and collect pure cell populations of the relevant NCAM^+^ cells out of tissue samples. Isolated pure NCAM^+^ cell populations were the most suitable starting material for downstream quantitative real-time PCR (qRT-PCR). Fig 4A and 4B represent renal tissues stained with anti-NCAM antibody prior to LCM procedure, and illustrate widespread NCAM expression in incipient IRF (Fig 4A) and scarce NCAM positivity in normal renal interstitium (Fig 4B). Fig 4C–4E illustrate tissues after LCM procedure. Statistically significant changes in the relative mRNA expression levels of NCAM isoforms have been revealed after applying qRT-PCR in the pure NCAM^+^ cell population. NCAM^+^ cells captured from incipient IRF significantly up-regulated NCAM^140kD^ isoform compared to NCAM^+^ cells in normal kidneys, p = 0.004 (Fig 4F). Nevertheless, mRNA expression levels of NCAM^120kD^ and NCAM^180kD^ isoforms were not changed significantly in comparison to normal kidneys (p = 0.750; p = 0.704; respectively).

### NCAM positive renal interstitial cells could share FGFR1, HE4 and α5β1 integrin

**Fig 5:**
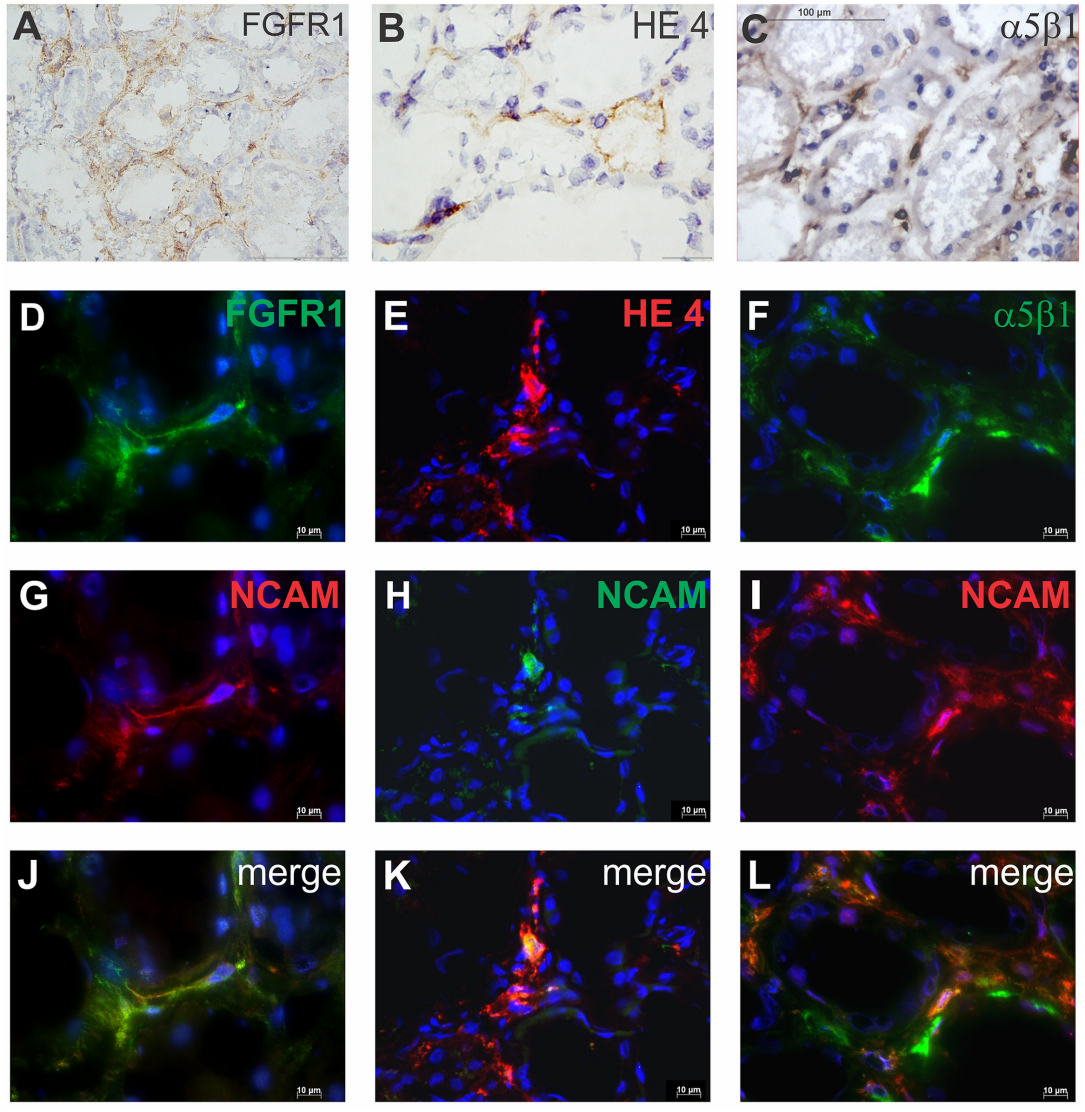
Expression of FGFR1, HE4 and α5β1 integrin on NCAM positive cells in normal renal interstitium. (**A**) FGFR1 expression on renal interstitial cells. (**B**) HE4 expression on renal interstitial cells. (**C**) Integrin α5β1 expression on renal interstitial cells. (A-C) cryostat sections, immunoperoxidase, x400; (**D**) FGFR1 expression. (**E**) HE4 expression. (**F**) Integrin α5β1 expression. (**G-I**) NCAM expression. (**J**) Coexpression FGFR1 and NCAM on the same interstitial cells. **(K)** Coexpression HE4 and NCAM on the same interstitial cell. **(L)** coexpression of α5β1 and NCAM on the same interstitial cells. (D-L) cryostat section, immunofluorescent labeling, x600; staining techniques are described in detail under Material and Methods.

Using immunoperoxidase staining, presence of few relevant markers for fibrosis has been investigated in renal interstitial cells: FGFR1 (Fig 5A), HE4 (Fig 5B), and α5β1 integrin (Fig 5C). Double immunofluorescence staining was applied to clarify whether NCAM^+^ renal interstitial cells could coexpress these markers.

Among 20 control kidneys, NCAM^+^ cells were detectable in 5 cases, usually one NCAM^+^ cell on magnification x400. Within these 5 cases, FGFR1 (Fig 5D), HE4 (Fig 5E) and α5β1 integrin (Fig 5F) could be detectable in the interstitium, and could be expressed in rare NCAM^+^ renal interstitial cells (Fig 5G–5I). Overlapping of these three molecules with NCAM is presented in Fig 5J–5L. However, great heterogeneity between NCAM^+^ renal interstitial cells was observed in different tissue samples, since not all NCAM^+^ cells coexpressed these molecules.

**Fig 6:**
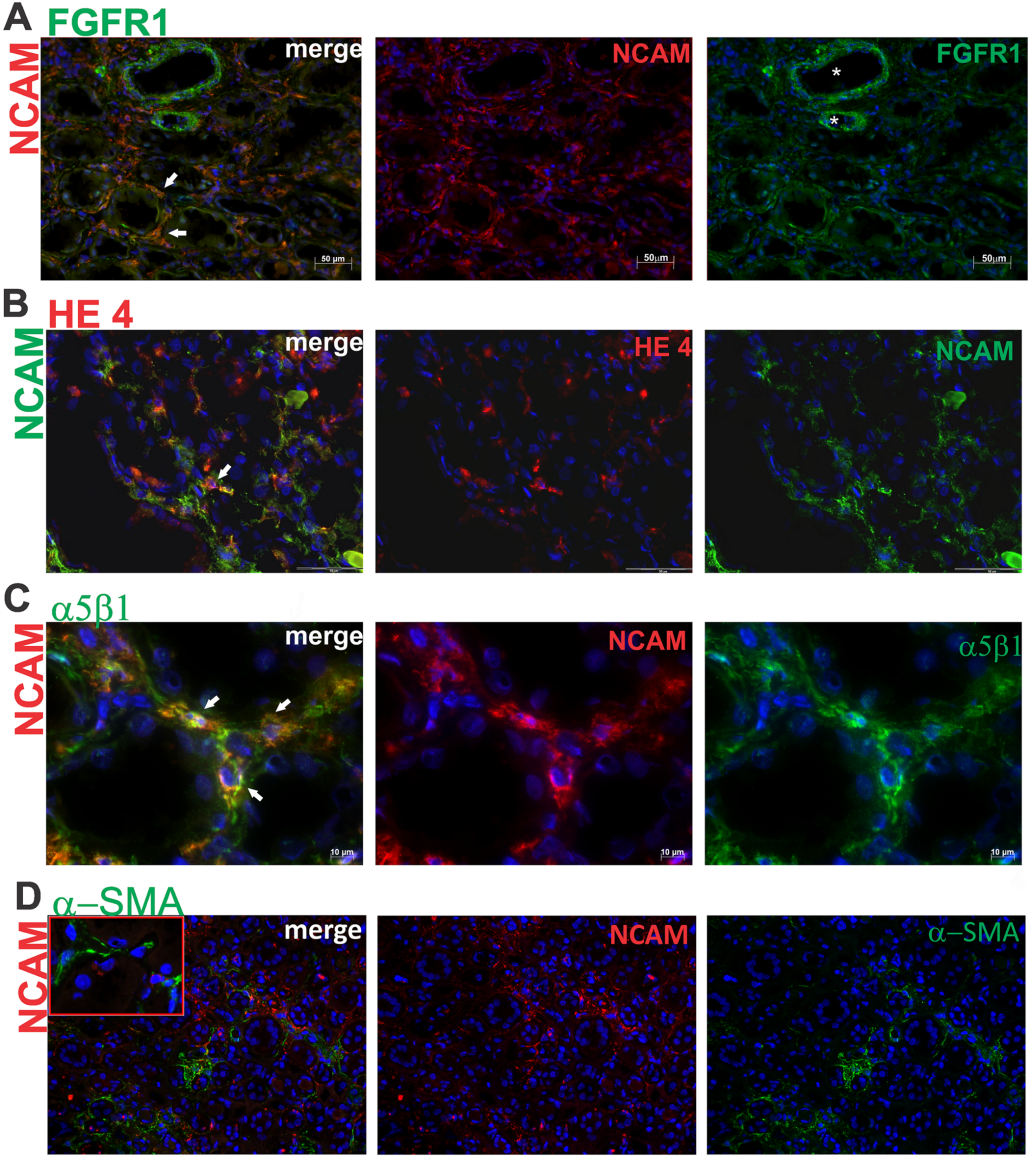
Expression of FGFR1, HE4 and α5β1 integrin on NCAM positive cells in incipient renal fibrosis. (**A**) Double immunofluorescent labeling of NCAM and FGFR1; merge of these two markers clearly shows that all NCAM+ cells coexpressed FGFR1 (white arrows); diffuse NCAM expression on interstitial cells; strong FGFR1 expression on bold vessels (white stars) and diffuse expression on interstitial cells; x200. (**B**) Double immunofluorescent labeling of NCAM and HE4; merge of NCAM and HE4 revealed single cells coexpressing both markers (white arrow); x400. (**C**) Double immunofluorescence labeling of NCAM and α5β1; merge of these two markers clearly shows co-expression of NCAM and α5β1 on renal interstitial cells in area of incipient fibrosis (white arrows); x600. **(D)** Double immunofluorescence labeling of NCAM and αSMA; merge of these two markers showed no overlapping of NCAM and αSMA on renal interstitial cells in area of incipient fibrosis, although areas of NCAM^+^ and SMA^+^ interstitial cells are close to each other; x100. Staining techniques are described in detail under Material and Methods.

Furthermore, in incipient IRF the increased NCAM^+^ renal interstitial cells could also coexpress FGFR1, HE4 or α5β1 integrin (Fig 6), with the great variability from case to case. For instance, in some cases with incipient IRF, slightly increased NCAM^+^/FGFR1^+^ renal interstitial cells were detected in comparison to controls, while other renal biopsy specimens with incipient IRF exhibited extensive, almost completely overlapping of NCAM and FGFR1 (Fig 6A). Less variability of NCAM and HE4 coexpression was observed, because in the most of the analyzed biopsies, only few NCAM^+^ cells shared HE4 molecule (Fig 6B), similar to control tissues. Nevertheless, compared to control renal tissue, overlapping of NCAM and α5β1 integrin was abundantly present in incipient IRF (Fig 6C). Double-immunofluorescent labeling for NCAM and αSMA revealed cells within the interstitium that expressed either NCAM or αSMA, whereby coexpression was not detected (Fig 6D).

### Relative mRNA expressions of molecules relevant for renal fibrosis

**Fig 7:**
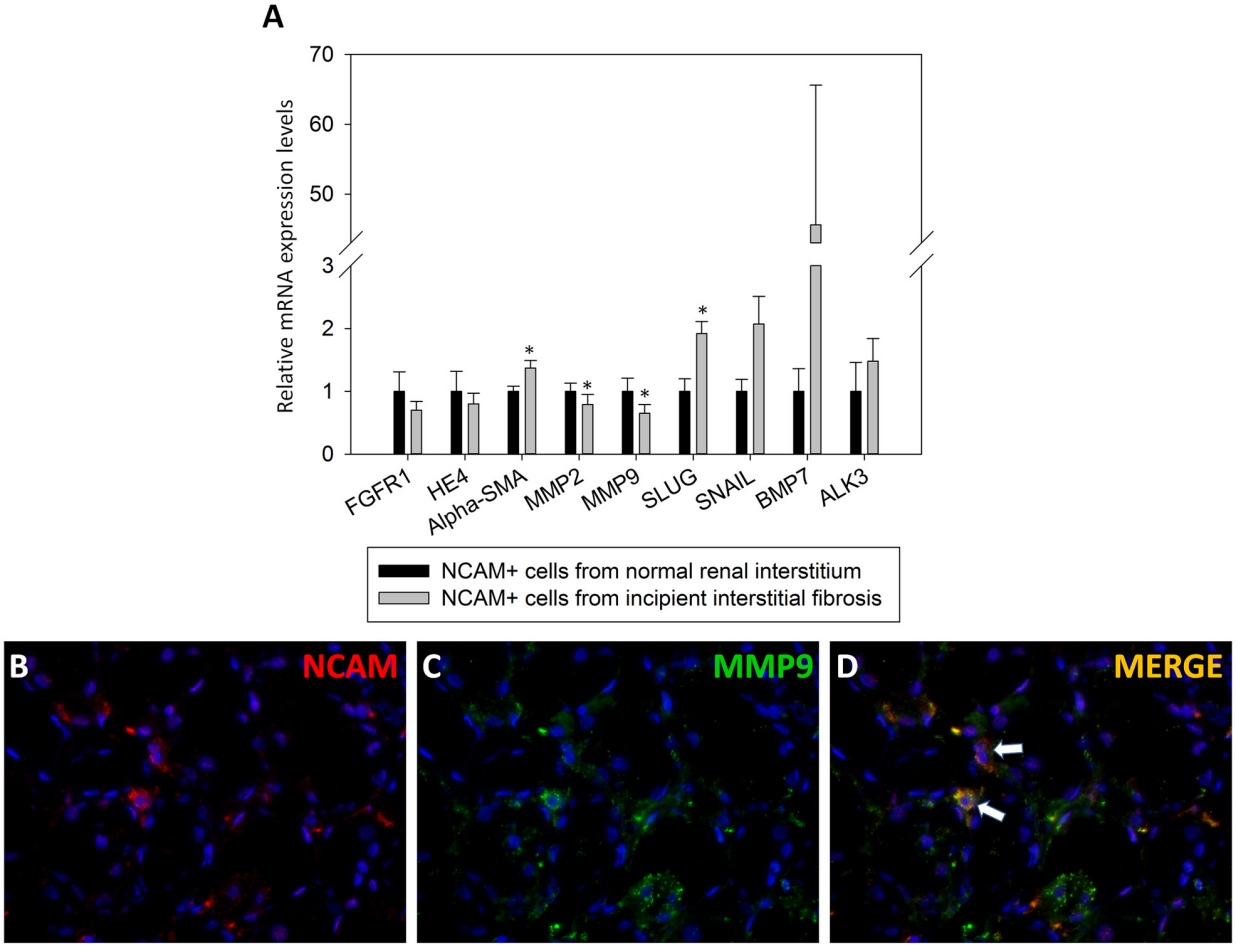
Differences in relative mRNA expression levels of molecules relevant for renal fibrosis among NCAM positive cells from normal and renal interstitium with incipient fibrosis, obtained by laser capture microdissection. **(A)** Relative mRNA expression levels of various molecules relevant for renal fibrosis; data are presented with mean values and standard error bars; *- indicate statistically significant difference, p<0.05; graph is made of mean values in order to unify variable presentations, although only αSMA, SLUG and ALK3 followed normal distribution; thus, some variables differed extremely among cases and consequently these data did not display normal distribution; due to influence of these extreme values on the mean value presented in the graph, bars are high but without statistical significance (such as BMP7); Student’s t test was used for variables with normal distribution both in control and kidneys with fibrosis: αSMA, SLUG and ALK3; due to high variability of other variables, exclusively in diseased kidneys, we applied nonparametric Mann Whitney U test to assess the difference in mRNA levels between controls and diseased kidneys; there were 6 samples (2 cases in triplicates) of control cases and 42 (14 cases in triplicates) samples of cases with incipient renal fibrosis. **(B)** NCAM positivity in peritubular incipient interstitial fibrosis shown on cryostat section, immunofluorescene, clone EP257Y, x400. **(C)** MMP-9 positivity in peritubular incipient interstitial fibrosis shown on cryostat section, immunofluorescence, clone 6-6B, x400. **(D)** arrows indicate the overlapping of NCAM and MMP-9 in interstitial cells.

While differences in mRNA expression levels detected by qRT-PCR between NCAM^+^ cells from fibrotic and non-fibrotic niche, captured by LCM, have not been significant in regard to FGFR1 and HE4 (p = 0.160; p = 0.151; respectively), significant increase in αSMA expression has been observed, p = 0.014 (Fig 7A). SLUG and SNAIL, known transcriptional factors which in turns up-regulate MMP-2 and -9 expressions, were also investigated in LCM captured NCAM+ cells. Here, we found significant mRNA over-expression of SLUG (p = 0.004) in NCAM^+^ cells situated in incipient IRF areas compared to NCAM^+^ interstitial cells in controls (Fig 7A). Due to highly variable SNAIL mRNA levels in interstitial NCAM^+^ cells of diseased kidneys, mRNA expression levels were not statistically different from controls (p = 0.704). MMP-2 and MMP-9 were even highly decreased (p = 0.028; p = 0.036; respectively) in NCAM^+^ cells laser captured from interstitium of the incipient IRF (Fig 7A), however, MMP-9 was detected on the protein level in rare cells within the renal interstitium (Fig 7B–7D). Since protective roles of BMP7 and its receptor ALK3 in renal fibrogenesis have been suggested and the role of interstitial NCAM^+^ cells was still not clarified, we further considered analyses of BMP7 and ALK3 expression levels in order to see whether NCAM^+^ cells have the ability to protect injured kidney from severe fibrosis. However, despite the presence of BMP7 and ALK3 over-expression in NCAM^+^ cells from kidney sections with incipient fibrosis, it did not reach statistical significance (p = 0.160; p = 0.424; respectively) since huge variability in mRNA levels were detected among samples, both in control and incipient IRF (Fig 7A). Mean mRNA levels with 95% Confidence Interval of the molecules relevant for renal fibrosis are presented in Table 3.

**Table 3:** Relative mRNA levels of molecules related to renal fibrosis in NCAM positive renal interstitial cells laser captured from normal and renal interstitium with incipient fibrosis.

	NCAM+ cells from normal renal interstitium [n = 6]		NCAM+ cells from incipient renal fibrosis [n = 42]	
	mean±SD	95% CI	mean±SD	95% CI
**FGFR1**	1.00±0.76	0.20–1.80	0.70±0.90	0.42–0.97
**HE4**	1.00±0.78	0.18–1.82	0.80±1.09	0.46–1.14
**αSMA**	1.00±0.19	0.80–1.20	1.37±0.76	1.13–1.60
**MMP2**	1.00±0.32	0.67–1.33	0.80±1.00	0.48–1.10
**MMP9**	1.00±0.52	0.45–1.55	0.65±0.88	0.38–0.93
**SLUG**	1.00±0.49	0.48–1.52	1.92±1.20	1.54–2.29
**SNAIL**	1.00±0.46	0.51–1.49	2.07±2.84	1.19–2.96
**BMP7**	1.00±0.88	0.08–1.92	45.60±129.55	5.23–85.97
**ALK3**	1.00±1.12	0.18–2.18	1.48±2.30	0.76–2.20

95% CI- 95% Confidence Inteval for Mean;

## Discussion

Progressive accumulation of extracellular matrix components leads to diffuse and abundant renal interstitial fibrosis causing renal failure. The major role in renal fibrosis belongs to activated fibroblasts, also known as myofibroblasts [23]. However, the origin of abundantly observed myofibroblasts within fibrotic areas is still debatable [14], especially considering that, in contrast to other human organs, normal renal tissue contains scarce fibroblasts [1, 24]. Previously it has been suspected that in incipient fibrosis, an increased number of NCAM^+^ cells appears without any clear clues regarding their origin, role and fate [2, 4], thus we tried to respond to these requirements at least in part.

In the present study, it has been statistically confirmed that an increased number of NCAM^+^ interstitial cells can be detected only in incipient IRF, while in severe fibrosis interstitial NCAM staining is almost absent. Similarly, an increased density of NCAM^+^ interstitial cells has been observed in early phase of repairing process after ischemic tubular injury in rats, with rapid decline a few days after injury [4]. Here we showed that increased interstitial NCAM positivity, appeared in incipient IRF, was independent of the underlying kidney diseases, although we cannot exclude that increase in number of NCAM^+^ interstitial cells was caused by hypoxia at least in some cases. In this setting, since EPO producing renal fibroblasts are expected to increase rapidly in response to tissue hypoxia [5, 21], double immunofluorescent labeling using EPO and NCAM antibodies was performed. Unfortunately, we were not able to detect any EPO and NCAM overlapping. However, considering that EPO expression levels are low, immunolabeling and even *in situ* hybridization analyses are already proved to be unconfident, and accordingly there is still a huge effort to visualize EPO producing cells *in vivo* [5, 21]. On the other hand, it is known that NCAM can be also expressed by NK cells of the innate immune system, but they were rarely present within renal interstitium, even in the cases of lupus nephritis, characterized with the recruitment of NK cells [25]. Hence, in incipient renal fibrosis an increased number of NCAM^+^ cell represented cell population different from NK cells. Altogether, it seems that NCAM expressing interstitial cells could have a regulatory role in the initial phase of kidney repair, and could represent a separate lineage existing in very low fraction in normal kidneys which proliferate in response to injury. Thus, NCAM^+^ interstitial cells could be only a portion of such heterogeneous interstitial cells in the human kidney [5, 26].

In order to clarify whether NCAM^+^ cells by itself differ in normal and interstitium with renal fibrosis, in this regard we primarily analyzed major NCAM isoforms and found the presence of all three major isoforms either in normal or in kidneys with fibrosis. However, for the first time, significant up-regulation of the NCAM^140kD^ isoform mRNA in incipient IRF has been detected, previously also described as the only major NCAM isoform up-regulated in ischemic cardiomyocytes [27]. Changes in NCAM^140kD^ expression levels were accompanied with almost unchanged levels of NCAM^120kD^ and NCAM^180kD^ transcripts in incipiently fibrosing and control kidneys, and the same pattern appeared in ischemic and normal cardiomyocytes [27]. After that discovery, Gattenlöhner and coworkers applied transcriptome analyses in order to reveal the role of NCAM^140kD^ overexpression and found its association with pro-apoptotic and anti-proliferative effects in cells which exhibit NCAM^140kD^ up-regulation [27]. Whether NCAM^140kD^ up-regulation in incipient renal fibrosis contributes to fibrosis or could have a protective role in that pro-fibrotic microenvironment, remains to be further clarified [28, 29].

Appreciating previous observations with regard to NCAM and FGFR1 interplay and their functional significance in the activation and proliferation of fibroblasts [6–9], it might be that detection of abundant NCAM/FGFR1 overlapping in some cases of incipient IRF can suggest possible progression to advanced stages of renal fibrosis, and it is tempting to speculate that inhibition of NCAM/FGFR1 interaction can ameliorate renal fibrosis which remains to be clarified in the future. Significant increase of α5β1 integrin has been previously detected in fibrotic kidneys [2, 13]. Here we found the presence of α5β1 integrin within the interstitial compartment of the kidney, with widespread NCAM overlapping merely in incipient IRF. Taking into account α5β1 integrin involvement in the activation of fibroblast, it could probably implicate the same fate of NCAM^+^/α5β1 integrin^+^ interstitial cells mainly detected in incipient IRF. In support of this observation, significant up-regulation of αSMA mRNA levels in NCAM^+^ cells from fibrotic kidney tissue was also found by qRT-PCR.

Important roles in extracellular matrix remodeling belong to metalloproteinases, especially MMP-2 and -9 which are able to degrade collagen [17], essential component of normal kidney interstitium but abundantly accumulated in renal fibrosis. Both MMP-2 and -9 were significantly down-regulated in NCAM^+^ interstitial cells situated within incipient IRF areas, despite significant up-regulation of SLUG mRNA level which is known transcriptional factors that usually regulates their expressions. These findings could implicate decreased capacity of NCAM^+^ cells in renal fibrosis to degrade extracellular matrix components compared to NCAM^+^ interstitial cells observed in normal kidney interstitium, potentially leading to maintenance and/or progression of a profibrotic microenvironment in the renal interstitium [17].

Nevertheless, since it has been shown that renal fibrosis could be at least ameliorated by BMP7 driving activation of its ALK3 receptor [20], we decided to explore them in NCAM^+^ interstitial cells. Although expression of BMP7 and ALK3 was not consistent within NCAM^+^ interstitial cells, neither captured from normal nor from incipient IRF areas, the presence of cases with increased BMP7 and ALK3 expressions in NCAM^+^ cells of incipient IRF could propose possibility to try with reversal of fibrosis by BMP7 and/or its homologue, as well as by ALK3 agonist administrations, similarly to previous attempts [20].

Prevention or even reversal of renal fibrosis requires better understanding of the complex network involved in fibrotic tissue response that leads to renal failure and kidney transplantation as the last therapy choice in the majority of patients who suffer from kidney diseases. Collectively our data reveal that increased NCAM^+^ cells observed in the renal interstitium of incipient fibrosis were heterogeneous and different from rare NCAM^+^ cells detected in normal kidneys, especially with regard to NCAM isoform switch concerning over-expression of NCAM^140kDa^ isoform in incipient renal fibrosis. Moreover, differences were also observed among tissues of renal fibrosis, on one hand suggesting that they share molecules involved in fibroblasts activation, with decreased capacity to be involved in degradation of excessively produced extracellular matrix components, but on the other hand with occasionally over-expressed BMP7 and ALK3 suggesting their protective function. Considering that up to now it could not be possible to obtain suitable animal model with selective NCAM knock-down in adult kidneys, since NCAM is an important molecule during embryonic kidney and brain development whose knock-down cause severe abnormalities, better *in vivo* characterization of NCAM positive cells can probably offer new opportunities to prevent and reverse natural disease course and ameliorate renal fibrogenesis as the common end stage of various renal diseases, especially considering that widespread NCAM expression revealed in the present study coincided exclusively with early phase of renal fibrosis independently of the underlying disease.
